# Validation of Malay Version of Body Self- Image Questionnaire-Short Form among Malaysian Young Adults

**DOI:** 10.21315/mjms2018.25.4.13

**Published:** 2018-08-30

**Authors:** Lim Chien Joo, Siti-Azrin Ab Hamid, Najib Majdi Yaacob, Suhaily Mohd Hairon, Kueh Yee Cheng, Mohamad Adam Bujang

**Affiliations:** 1 Unit of Biostatistics and Research Methodology, School of Medical Sciences, Health Campus, Universiti Sains Malaysia, 16150 Kubang Kerian, Kelantan, Malaysia; 2 Department of Community Medicine, School of Medical Sciences, Health Campus, Universiti Sains Malaysia, 16150 Kubang Kerian, Kelantan, Malaysia; 3 Clinical Research Centre, Sarawak General Hospital, Ministry of Health, Malaysia

**Keywords:** body image, questionnaire, validation

## Abstract

**Background:**

Body self-image questionnaire-short form (BSIQ-SF) is developed to measure body image perceptions. Due to the cultural, language and environmental differences between western and eastern population, the validity and reliability need to be established. The aim of this study was to determine validity and reliability of Malay version BSIQ-SF.

**Methods:**

A cross-sectional study involved web-based survey was employed. Exploratory factor analysis (EFA) and confirmatory factor analysis (CFA) was performed using SPSS version 22 and Mplus 7.3.

**Results:**

There were 688 young adults in Malaysia with mean age of 23.67 (SD = 0.188) and mean body mass index (BMI) of 23.34 (SD = 0.27) participated in the study. Exploratory factor analysis performed and the number domains reduced from nine to four, namely ‘Negative Affect’, ‘Attractiveness Evaluation’, ‘Physical Functionality Awareness’ and ‘Height Dissatisfaction’. CFA further confirmed the structure of the model with adequate goodness-of-fit values [CFI = 0.927, TLI = 0.913, SRMR = 0.075, RMSEA = 0.053 (95% CI: 0.047, 0.060)].

**Conclusion:**

The revised 21-item of the Malay version BSIQ-SF was a valid and reliable instrument to measure body image perceptions among Malaysian young adults.

## Introduction

Body image has a multidimensional construct containing both positive and negative features with perceptual, attitudinal and behavioural aspects (1, 2). Positive body image is being happy in our own body, being comfortable most of the time with our physical appearance and feels good about ourselves. Individual who has positive body image accept the way they look and feel positive about their appearance most of the time. Although their appearance may not match the ideals of the society, they learned to be proud of their body image.

Negative body image, or body dissatisfaction, develops when an individual feel that his or her body does not meet the standard of family, social or media ideals. Individuals who have negative body image may not see themselves as they truly are, and are often very dissatisfied.

The concern of body image is increasing and social psychology demonstrated the impact of an individual’s physical appearance on how others perceive and interact with him or her. Studies have shown individuals with unattractive appearance receive negative evaluations from their peers and reduced social contact (3, 4).

People who are dissatisfied with their body image tend to have lower self-esteem than others, affecting their social life as they are less confident and feel uneasy to meet new social groups and therefore, quality of life affected. Social isolation and low self-esteem affect the personality and emotional stability of an individual, especially young adult who might not be good enough to manage their emotion and thinking. Relationship with body image has been found in young adults with emotional instability, depression, anxiety or substance abuse (5).

Furthermore, body dissatisfaction brings significant negative consequences to the health status of an individual in the form of excessive dieting (6) and eating disorders (7). Excessive dieting and eating disorders impact the health status of an individual by losing weight or not obtaining proper nutrition, consequently leading to poorer health such as anaemia, tooth decay, low blood pressure and others.

### Body Self Image Questionnaire (BSIQ)

BSIQ was first developed in the year 1999 (8) to measure body image in young adults. The development of this questionnaire involved the collection of data in three separate studies. In Study 1, open-ended questions were developed from a review of body image literature and review of instruments available to measure body related traits. The aim of Study 1 was to develop statement items for BSIQ. While for Study 2 and Study 3, exploratory factor analyses and item-subscale correlations were used to guide revisions to the questionnaire.

The results of Study 2 and Study 3 revealed nine factors consisting Overall Appearance Evaluation, Fatness Evaluation, Health/Fitness Evaluation, Negative Affect, Health/Fitness Influence, Social Dependence, Investment in Ideals, Attention to Grooming and Height Dissatisfaction. This questionnaire consisted 51 items with internal consistency reliabilities for the subscales ranged from 0.68 to 0.92. Factor loading in Study 3 supported the 9-factor structure, with one exception. There was some ambiguity existed in 2 subscales (Negative Affect and Social Dependence), whose factor loadings suggested the possibility of a single factor.

The preliminary results showed that BSIQ is a validated and reliable instrument by offering a multidimensional measure of body image. It was developed using a comprehensive, multistage process; nevertheless, further research is needed to build on this evidence using confirmatory factor analyses and external validity evidence. Further research was conducted in the year 2005 to support the validity of BSIQ (9). The purpose of the study was to simplify the original version of BSIQ from 51 items to 27 items.

Body self-image questionnaire-short form (BSIQ-SF) is a validated and reliable measurement tool offers a theoretically and empirically supported questionnaire to measure nine dimensions of body image with three item per factor. The BSIQ-SF provides practical advantages over the 51-item version in large sample structural equation modelling studies as well as reduce response burden, which has been proposed to lead to poorer response rate, lower completion and reduced data quality (10).

Currently, there is no published instrument that measures the measure body image perceptions in the Malay language. It is important to understand that it is crucial for the researcher to explore the issues related to body image in the local context. Therefore, the aim of this study was to translate the BSIQ-SF into the Malay language and to examine its reliability and validity using exploratory and confirmatory factor analysis.

## Method

### Participants

Participants were 688 young adults (188 respondents for Phase 1 exploratory factor analysis (EFA) and 500 respondents for Phase 2 confirmatory factor analysis (CFA)) in Malaysia aged between 18 to 35 years (mean = 23.67, standard deviation (SD) = 0.19), with a mean body mass index (BMI) of 23.34 (SD = 0.27). The majority of them were single (81.6%), while 17.8% of them were married and only 0.6% of them divorced. Around 79.9% of the participants lived in Peninsular Malaysia while the rest lived in East Malaysia. In term of race, 82.3% of the participants were Malay, followed by Chinese 10.0%, Indian 1.3% and natives from East Malaysia (6.3%). Educational level of the participants was as follows: undergraduate (37.5%), secondary (23.8%), diploma (16.5%), pre-university (10.8%), certificate (5.2%) and postgraduate (4.6%). Only a minority of them had an informal education (0.9%) or had their education until primary school only (0.6%).

According to Tabachnick et al*.*, the required sample size for EFA is 150 subjects (11). While Hair et al. suggested that sample size calculation for CFA should be based on model complexity. A sample size of 500 or more should be recruited for a model with more than six constructs, less than three items and low communalities (12). The BSIQ-SF comprised of 9 factor with 3 items under each factor, therefore a sample size of 500 were recruited.

### Design

The study was a cross-sectional study involved web-based survey. The study had two phases: Phase 1 EFA and Phase 2 CFA. Source population were the young adults in Malaysia which the participants were recruited using snowball sampling method. The undergraduate students from Universiti Sains Malaysia Health Campus were approached as the initial seed of the study. The inclusion criteria include: participants aged between 18–35 years old, able to read and understand Bahasa Malaysia, and available at time of data collection; while the exclusion criteria include: young adults who did not have internet access and had answered the questionnaire before.

## Materials

The instrument used in this study was Body Self-Image Questionnaire-Short Form (BSIQSF). BSIQ-SF consisted nine factors namely ‘Overall Appearance Evaluation’, ‘Health Fitness Influence’, ‘Investment in Ideals’, ‘Health-Fitness Evaluation’, ‘Attention to Grooming’, ‘Height Dissatisfaction’, ‘Fatness Evaluation’, ‘Negative Affect’ and ‘Social Dependence’, with three items in each subscale. Responses were recorded in form of 5-point Likert scale ranged from ‘Not at all True of Myself’, ‘Slightly True of Myself’, ‘About Halfway True of Myself’, ‘Mostly True of Myself’, and ‘Completely True of Myself’. Two cohorts were recruited for factorial validity and cross validity testing of the short form of the questionnaire. Factorial validity showed satisfactory results (*X*^2^ = 2210.19, CFI = 0.93, NNFI = 0.92, RMSEA = 0.04) with meaningful item loadings in both samples, factor loading for cohort 1 ranged from 0.62 to 0.96 while the factor loading for cohort 2 ranged from 0.55 to 0.94. The model fit was achieved without resorting to correlated errors or cross loading (*X*^2^ = 2427.54, RMSEA = 0.41 (90% CI = 0.039–0.043), CFI = 0.927, NNFI = 0.923).

### Procedure

Approval was granted from Human Research Ethics Committees (HREC) of Universiti Sains Malaysia (USM). The main language spoken among students in Malaysia is Malay, therefore we translated the BSIQ-IF from the original English version to Malay and named this Malay version BSIQ-SF. Permission has been obtained from the original author to utilise and translate the questionnaire. The translation process was adapted from World Health Organization (2018) for the Management of Substance Abuse (13). Forward-backward translation was done and reviewed by experts, investigators and translators involved. Upon agreement, the questionnaire was pre-tested for face validity and content validity.

Participants were approached and asked for willingness to take part in the study. Implied consent was used when participants ticked on a consent statement before they can answer the questionnaire online. The respondents who agreed to take part were asked to complete the survey form and submit it online. They were encouraged to share the link to their peers who fulfilled the inclusion and exclusion criteria. There were no missing data in this study as all the questions were set to be mandatory to answer before they can proceed to the next section.

### Statistical Analysis

Data were gathered in a response spreadsheet once the participants submitted the survey form. EFA was conducted using IBM SPSS statistics Version 22 and CFA was conducted using Mplus version 7.3.

### Exploratory Factor Analysis (EFA)

**Figure 1:**
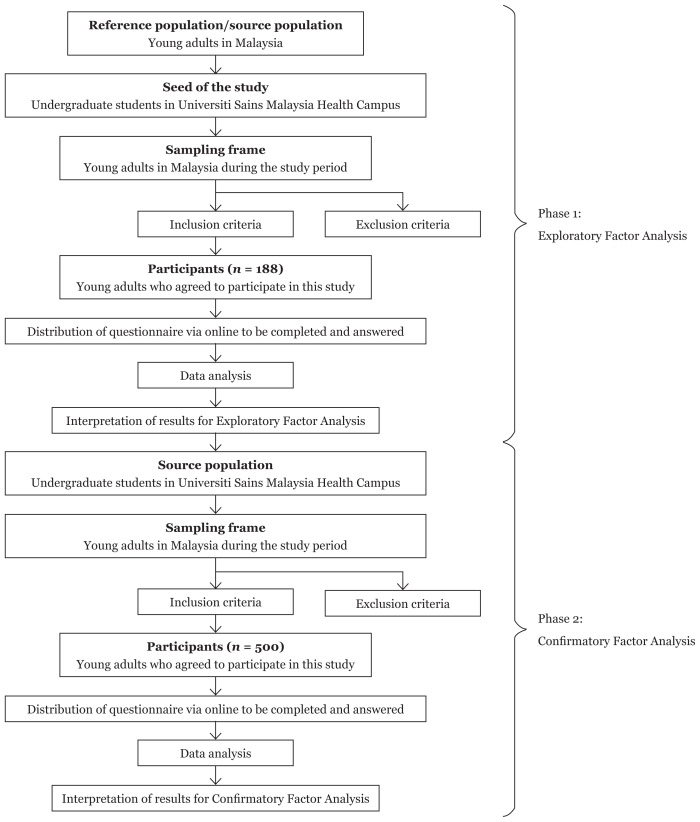
Flowchart of study

Data were analysed using EFA to determine the underlying relationship between the measured variables. Univariate normality was tested via histogram while positive definiteness of data matrix was evaluated using principal component analysis. All the eigenvalues were more than zero for data matrix to be positive definite. Multicollinearity assessment was done via tolerance and variance inflation factor (VIF) and multivariate collinearity is highly unlikely when VIF value was less than 10 (14). KMO and Bartlett’s test was performed and according to Williams et al. (15), KMO index of 0.50 is considered suitable for factor analysis while Barlett’s test of Sphericity should be significant for factor analysis to be suitable. Factor rotation Promax and Principal Axis Factoring (PAF) was used for factor extraction and the number of factors to be extracted was based on eigenvalues and scree plot. The eigenvalue of 1 indicates factor is worthwhile to be extracted (16), while for scree plot, the last substantial decline in the plot (elbow) was observed and the number of factors to be extracted was the number of dots above the ‘elbow’ (17). Item removal was determined based on factor loading and communalities. The cut off for factor loading was set at 0.4 and communalities was set at 0.3. The flow of statistical analysis was presented in Figure 1.

### Confirmatory Factor Analysis (CFA)

CFA, a multivariate analysis to examine how well the measured variables are representing their constructs, was used to assess the validity and reliability of the translated questionnaire. Multivariate normality and presence of multivariate outliers were checked using Mahalanobis distance plot. Maximum likelihood robust estimator (MLR) was used in the measurement of model validity. Model fit of CFA depends on absolute fit by the standardised root mean square residual (SRMR), parsimony correction fit index by the root mean square of approximation (RMSEA), comparative fit index (CFI) and Tucker-Lewis Index (TLI). SRMR of ≤ 0.08 indicates good fit while RMSEA of ≤ 0.08, CFI and TLI between 0.90–0.94 indicates the model is reasonably fit (14). If the model does not fit the data, necessary modification to improve model fit were done by removing items with low factor loading, high standardised residuals and high modification index. Modifications were done until the model was reasonably fit as well as theoretically sound. Construct reliability (CR) was checked by Raykov’s procedure (18) where recommended value for CR was 0.7 which suggest good reliability whole 0.6 was acceptable provided that other indicators of a model’s construct validity were good (19). The flow of statistical analysis was presented in Figure 1.

## Results

### Exploratory Factor Analysis

EFA was started with assumptions checking. All the data in the model were positive definite by having all the eigenvalues more than zero. Multicollinearity was highly unlikely as there was no value of VIF exceeding 10. KMO index of 0.870 and Bartlett’s test value of < 0.001 indicated that data were worthwhile for factor analysis. Five factors were extracted from the initial model and no items were dropped as all of the items had communalities and factor loading more than 0.3. The model was finalised with four factors after taking considerations on the factor loading loaded on each factor. Items whose highest loading was on the same factor were grouped. The extracted four factors in the model were renamed: Negative Affect, Attractiveness Evaluation, Physical Functionality Awareness and Height Dissatisfaction. The final model consisted four latent variables with 27 items (Table 1).

### Confirmatory Factor Analysis

Assumptions checking was done before CFA as well. Chi-Square versus Mahalanobis distance plot was plotted and assumptions of multivariate normality were not met. Mplus software was used to examine the normality of the model and two-sided multivariate skew test of fit and results showed significant *P*-value. Since the data does not meet the normality assumption, the remedy for this was the use of unbiased estimator, robust maximum likelihood (MLR) estimator in Mplus software for CFA analysis. All the data were positive definite and multicollinearity was highly unlikely as VIF value does not exceed 10.

The initial model revealed poor fit of data for 27 items. Item 27 and Item 23 were removed due to low loading factor of lower than 0.4; nonetheless, model fit was still not within the acceptable threshold value.

Covariance among items’ residual (within factor) were checked and added iteratively. A total of six items’ residual were added to the model: Item 25 with Item 16, Item 19 and Item 18, Item 22 and Item 19, Item 25 and Item 7, Item 16 and Item 7, Item 17 and Item 16. However, the threshold value of the fit indices was not met yet.

The model was further examined. Item 2, Item 10, Item 12 and Item 5 were removed iteratively due to high standardised residual and suggested in Modification Indices to correlate with items among different factors. The model fit improved substantially and the fit indices were within the acceptable threshold value (Table 2).

Composite reliability was computed for each factor. The factor Height Dissatisfaction demonstrated highest composite reliability of 0.857 (0.830, 0.883), followed by Negative Affect 0.846 (0.819, 0.873), Physical Functionality Awareness 0.747 (0.704, 0.790) and Attractiveness Evaluation 0.736 (0.690, 0.782).

Total of six items were removed and six covariance among item’s residual were added for final model and the model demonstrated satisfactory model fit [CFI = 0.927, TLI = 0.913, SRMR = 0.075, RMSEA = 0.053 (95% CI: 0.047, 0.060)]. Overall, the final measurement model demonstrated a good validity and construct reliability.

## Discussion

The original BSIQ-SF consisted nine latent factors with 27 items namely: Overall Appearance Evaluation, Health Fitness Influence, Investment in Ideals, Health Fitness Evaluation, Attention to Grooming, Height Dissatisfaction, Fatness Evaluation, Negative Affect and Social Dependence. However, during EFA process and consideration on the factor loading, communalities, reviewed the meaning of every item and discussed with experts, the items were re-grouped into four factors: Attractiveness Evaluation, Negative Affect, Physical Functionality Awareness and Height Dissatisfaction (Table 3).

Items under factor Negative Affect include Item 1, Item 5, Item 7, Item 8, Item 14, Item 16, Item 17, Item 23, Item 25, Item 26 and Item 27. These items were adopted from factor Overall Health Evaluation, Social Dependence, Negative Affect and Fat Evaluation from original nine factor measurement model. Factor Social Dependence and Negative Affect demonstrated a degree of overlap when the original author first developed the questionnaire, thus it is not surprising that these factors were grouped under one factor when EFA performed (8). Factor Negative Affect incorporates the influence of body image on negative emotional well-being, which consequently leads to body dissatisfaction and misperceptions of body image. Misperceptions of body image greatly impact the health status of a person, physically, physiologically and emotionally. Altered body image perception has gained the attention of health care professionals as an important public health care issue (20). Therefore, it is essential to be included into the model when we measure body image perception.

Items under factor Attractiveness Evaluation include Item 4, Item 13, Item 18, Item 19, Item 21 and Item 22. Items were adopted from factor Overall Health Evaluation, Health Fitness Evaluation and Attention to Grooming. All the items under these factors incorporate evaluative components, in both appearance and health fitness aspects. Body image satisfaction is associated with attractiveness evaluation when individuals evaluate and compare their physical attributes with others, regardless to whom they compare. Physical attractiveness greatly impacts one’s social life; taking example attractive people are more likely to get hired or welcomed by others. Furthermore, attractive people are more concerned about their body image and constantly monitor their physical appearance to stay attractive. Domain Attractiveness Evaluation measures an individual’s perception on how attractive he/she is physically and physiologically. Individual who perceived him/ herself as attractive most likely has positive body image.

Items under factor Physical Functionality Awareness include Item 2, Item 3, Item 9, Item 10, Item 11, Item 12 and Item 20. These items were originally adopted from factor Investment in Ideals, Attention to Grooming, and Health Fitness Influence. Physical functionality is included as one of the aspects measuring body image as body image is associated with physical well-being as well. Furthermore, research also found that negative body image can restrain a person from participating in physically active leisure activities (21). Poor body image is common among young adults as dieting may slow down cognitive performance as well as physical activity behaviours. In other words, individuals with high physical functionality awareness will be more active in involving themselves in physical activities which ultimately promotes positive body image.

Items under factor Height Dissatisfaction are Item 6, Item 15 and Item 24. This factor is stable and consistent since the questionnaire first developed to appear as a distinct factor alone. Element height has constantly been included in measuring body dissatisfaction, and height dissatisfaction is common.

CFA was performed to assess the validity of the structural theory and identify a valid measurement model. MLR was commonly used as fitting function for structural equation models in CFA, assuming that variables in the model fulfilled the multivariate normality assumption (22). MLR was robust against moderate violations of assumption such as un-modelled heterogeneity (23) and able to accommodate non-normality data. This study employed MLR estimation method as the items were not multivariate normal (24).

Findings of this study yield different number of domains compared with the BSIQ-SF, most probably due to the cultural difference between eastern and western country, signifying variant in the understanding on different set of translated questionnaire for different population. The final model consisted 21 items with four factor model fits well after re-specification. In the re-specification process, six items (22.2%) out of 27 items were dropped and six items’ residual were added to the model.

The final model indicates a reasonably good model fit as all the fit indices were within a good and acceptable range. CF fit were used to assess the overall fit and a p-value of 0.203 indicates good model fit. RMSEA is a parsimony-adjusted index and value closer to zero represent good fit. The RMSEA of this study was 0.053, indicating reasonably fit as proposed by Kline (2011) (14). The CFI and TLI compares the fit of the target model to the fit of an independent (null) model, and the results of this study were within the range between 0.90–0.94, showing reasonably fit as well. The validity was further confirmed by SRMR by looking at the square root of the difference between the residuals of the sample covariance matrix and the hypothesised model by showing a result of 0.075 which was lower than the cut off value 0.08 (14).

The present study confirmed the validity for the four factor structure measurement model based on CF fit, RMSEA, SRMR, CFI and TLI, providing a foundation for future study of this instrument as a culturally appropriate tool to investigate body image perception among Malaysian young adults.

Two types of reliabilities were computed in the present study: Cronbach alpha in EFA phase and composite reliability based on formula proposed by Raykov and Marcoulides (2015) in CFA phase (25). Cronbach alpha was provided in EFA phase to allow comparison between the present study with other study. It was reported that the internal consistency reliabilities of the original BSIQ ranged from α = 0.68 to α = 0.92 (8). In our study, the Cronbach alpha ranged from α = 0.797 to α = 0.850. CR based on Raykov and Marcoulides formula was commonly used to determine the reliability of the factors after analysis of CFA (26–28) because it accounted for error covariance, providing a less bias estimate of reliability than Cronbach alpha (23). The CR produces the estimates of true reliability with confidence interval, allowing the empirical assess and overcome some of the limiting assumption of coefficient alpha (25, 29) in CFA study (26).

The original author mentioned in their study that participants recruited were university students where the majority of them (88%) were from within the same state, and there was a disproportionate number of students from each grade level. Therefore, generalisation to young adults other than students and other regions should be taken with caution (8). In the current study, participants recruited were not limited to university students only but young adults aged 18 to 35, and they were from different states in Malaysia. There were slightly more female participants (66.4%) compared to male participants (33.6%) in this study, most probably due to the reason that female has higher willingness to respond and participate in the survey compared to male. Therefore, this validated Malay version of questionnaire is sufficient to represent young adults in Malaysia.

Previous study also mentioned invariance study across gender was not performed as female were overrepresented in the study (8). Further invariant analysis should be taken to investigate gender differences in the factor structure for this translated and validated Malay version body self-image questionnaire, too.

## Conclusion

In conclusion, the findings showed that the revised 21-item of the Malay version BSIQ-SF was reliable and valid among the young adults in Malaysia who were surveyed in this study. The results for EFA retained all the items and re-grouped them into four factors. The final model for CFA showed good model fit, valid and reliable after removing six items. However, improvements are needed for future research using Malay version BSIQ-SF to attain more accurate results for different study populations and age groups. This study developed the Malay version BSIQ-SF, which can be used in future research examining perception of body images, where the Malay language is the main spoken language among the study participants.

The final version of the Malay version BSIQ-SF is shorter than the original version, with 51 items and nine factors on perception of body images. This might be valuable, given that a criticism of the 51-item BSIQ has been that it might be too long for use with a range of the population, who might get tedious while completing it.

## Figures and Tables

**Table 1:** Item factor loading and communalities for EFA and CFA

Item	Question	EFA		CFA	
	
		Factor loading	Cronbach alpha	Factor loading	Composite reliability
Negative Affect					
Item 1	I think my body is unattractive	0.547	0.844	0.558	0.846 (0.819, 0.873)
Item 5	I compare my body to people I’m close to (friends, relatives, etc.)	0.480		-	
Item 7	I think my body looks fat in clothes	0.669		0.734	
Item 8	My naked body makes me feel sad	0.656		0.668	
Item 14	Being around good-looking people makes me feel bad about my body	0.630		0.726	
Item 16	My body is overweight	0.699		0.679	
Item 17	I feel depressed about my body	0.844		0.815	
Item 23	I’m more aware of my body when I’m in social situations	0.404		-	
Item 25	I wish I were thinner	0.519		0.606	
Item 26	Most days I feel bad about my body	0.878		0.773	
Item 27	I spend time making my appearance more attractive	0.619		-	

Attractiveness Evaluation					
Item 4	My overall fitness level is high	0.360	0.797	0.681	0.736 (0.690, 0.782)
Item 13	My body is healthy	0.437		0.725	
Item 18	I’m usually well-dressed	0.449		0.459	
Item 19	My body looks good	0.816		0.572	
Item 21	I care about how well-shaped my legs are	0.329		0.416	
Item 22	My body is in shape	0.921		0.743	

Physical Functionality Awareness					
Item 2	How well my body is functioning influences the way I feel about my body	0.495	0.850	-	0.747 (0.704, 0.790)
Item 3	Having a well-proportioned body is important to me	0.908		0.558	
Item 9	I pay careful attention to my face and hair, so that I will look good	0.537		0.556	
Item 10	I look good in clothes	0.571		-	
Item 11	I feel better about my body when I’m fitter	0.794		0.775	
Item 12	Body size matters to me	0.642		-	
Item 20	The way I feel about my body improves when I exercise regularly	0.682		0.668	

Height Dissatisfaction					
Item 6	I’ve often wanted to be taller	0.787	0.803	0.809	0.857 (0.830, 0.883)
Item 15	I wish I were a different height	0.791		0.869	
Item 24	If I were a different height, I’d like my body better	0.660		0.762	

**Table 2:** Fit indices for Body Self-Image Measurement Model among young adults–CFA

Fit indices	Cut off value	Source	Model results
CF fit	*P-*value > 0.05		0.203
RMSEA (90%CI)	< 0.05, model is good fit ≤ 0.08, reasonably fit, and < 0.10 indicate poor fit	Kline (14)	0.053 (0.047, 0.060)
SRMR	≤ 0.08 indicates a good fit		0.075
CFI	≥ 0.95, good fit		0.927
TLI	Between 0.90–0.94, reasonably fit	Kline (14); Wang and Wang (18)	0.913

**Table 3:** Items, scoring and remarks for each domain for Malay version BSIQ-SF

Domain	Item	Scoring	Remarks
Negative Affect	Item 1	1–40	Measures the influence of body image on negative emotional well-being. Higher score indicates higher degree of body dissatisfaction.
	Item 2		
	Item 3		
	Item 4		
	Item 5		
	Item 6		
	Item 7		
	Item 8		

Attractiveness Evaluation	Item 9	1–30	Measure self-evaluation in appearance and health fitness aspects. Higher score indicates higher body satisfaction.
	Item 10		
	Item 11		
	Item 12		
	Item 13		
	Item 14		

Physical Functionality Awareness	Item 15	1–20	Measure the awareness of an individual towards his/her own physical functionality. Higher score indicates higher awareness to maintain good physical functionality.
	Item 16		
	Item 17		
	Item 18		

Height Dissatisfaction	Item 19	1–15	Measure the degree of dissatisfaction on height. Higher score indicates higher dissatisfaction towards one’s own height.
	Item 20		
	Item 21		

Scoring is based on the summation from all the items under the domain.

Preferably all the items have to be answered, otherwise single imputation using mean can be done to replace missing scores (recommended: Negative Affect: at most three missing scores; Attractiveness Evaluation and Physical Functionality Awareness: at most two missing scores; Height Dissatisfaction: at most one missing score)
